# Diet and human reproductive system: Insight of omics approaches

**DOI:** 10.1002/fsn3.2708

**Published:** 2022-03-21

**Authors:** Xiaoling Ma, Luming Wu, Yinxue Wang, Shiqiang Han, Marwa M. El‐Dalatony, Fei Feng, Zhongbin Tao, Liulin Yu, Yiqing Wang

**Affiliations:** ^1^ The First Hospital of Lanzhou University The First School of Clinical Medicine Lanzhou University Lanzhou China; ^2^ Gansu International Scientific and Technological Cooperation Base of Reproductive Medicine Transformation Application Key Laboratory for Reproductive Medicine and Embryo Lanzhou China; ^3^ Linxia Hui Autonomous Prefecture Maternity and Childcare Hospital Linxia China

**Keywords:** amino acid metabolism, male and female fertility, nutrigenomics, placental transfer, reproductive health

## Abstract

Nutrition and lifestyle have a great impact on reproduction and infertility in humans, as they are essential for certain processes such as implantation, placental growth, angiogenesis, and the transfer of nutrients from the mother to the fetus. The aim of this review is to provide the interconnection between nutrition and reproductive health through the insight of omics approaches (including metabolomics and nutrigenomics). The effect of various macronutrients, micronutrients, and some food‐associated components on male and female reproduction was discussed. Recent research work was collected through database search from 2010 to 2020 to identify eligible studies. Alterations of metabolic pathways in pregnant women were deliberated with an emphasis on different strategies of lifestyle and dietary interventions. Several nutritional methods, which are important for embryonic and child neurological development, nutritional supplements to lactation, and improved gestational length along with birth weight have been emphasized. Considerable advances in omics strategies show potential technological development for improving human reproductive health.

## Graphical abstract

In this review, we clarify the importance of nutrition and dietary substrates in the reproductive health of humans. Omics (including metabolomics and nutrigenomics) have been employed to report the nutrients’ impact on physiological functions at the molecular level and to explain how diet and specific nutrients interact with genes, proteins, or metabolites to influence metabolic phenotypes and disease outcomes. Novel nutritional methods and compositions containing essential nutrients were explored to enhance embryonic, fetal, and child neurological development, afford nutritional supplementation to lactation, and improve gestational length and birth weight.

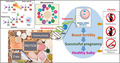

## 1 BACKGROUND

**FIGURE 1:**
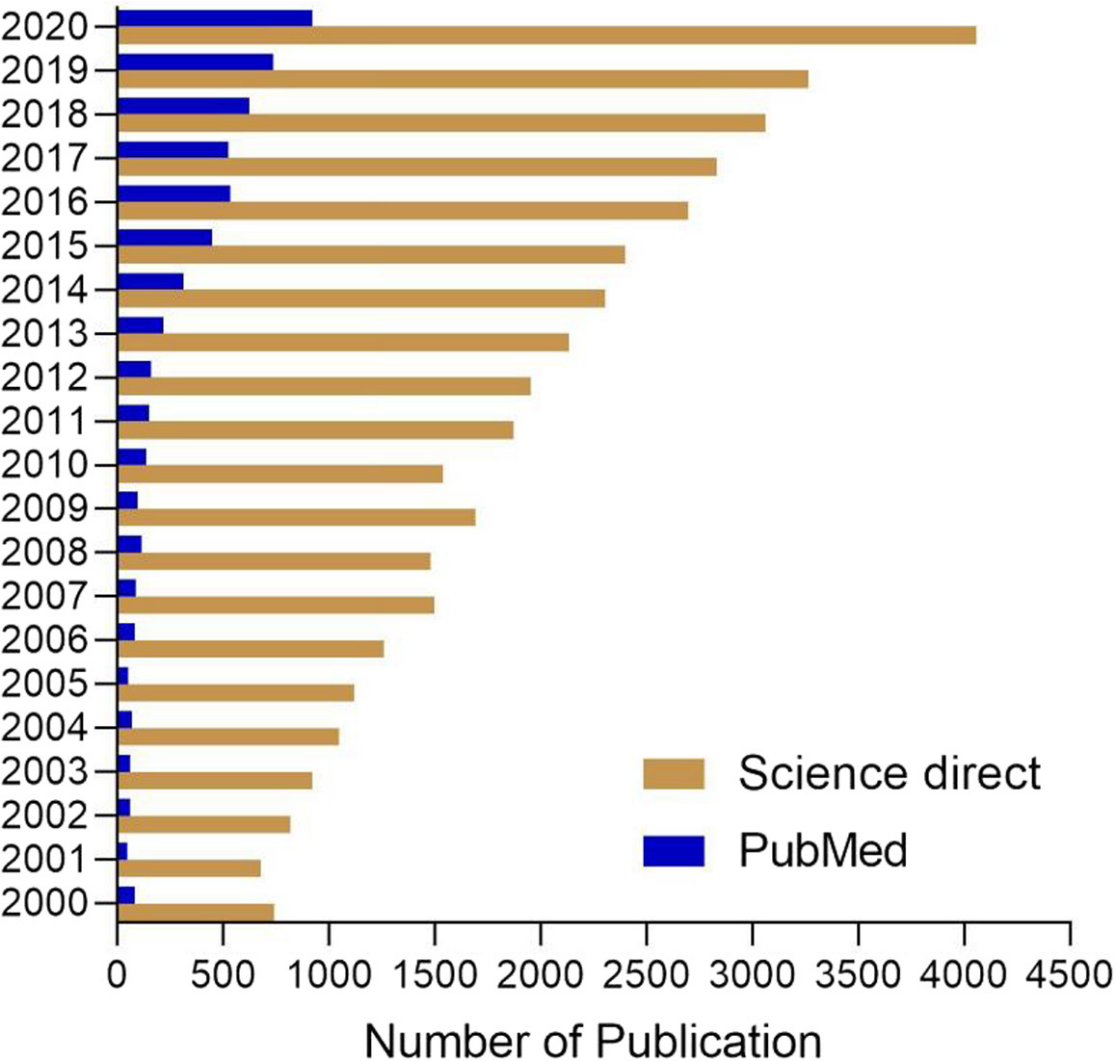
Number of publications reporting the relation between nutrition and human reproductive health. Data were obtained from ScienceDirect and PubMed from 2000 to 2020

**FIGURE 2:**
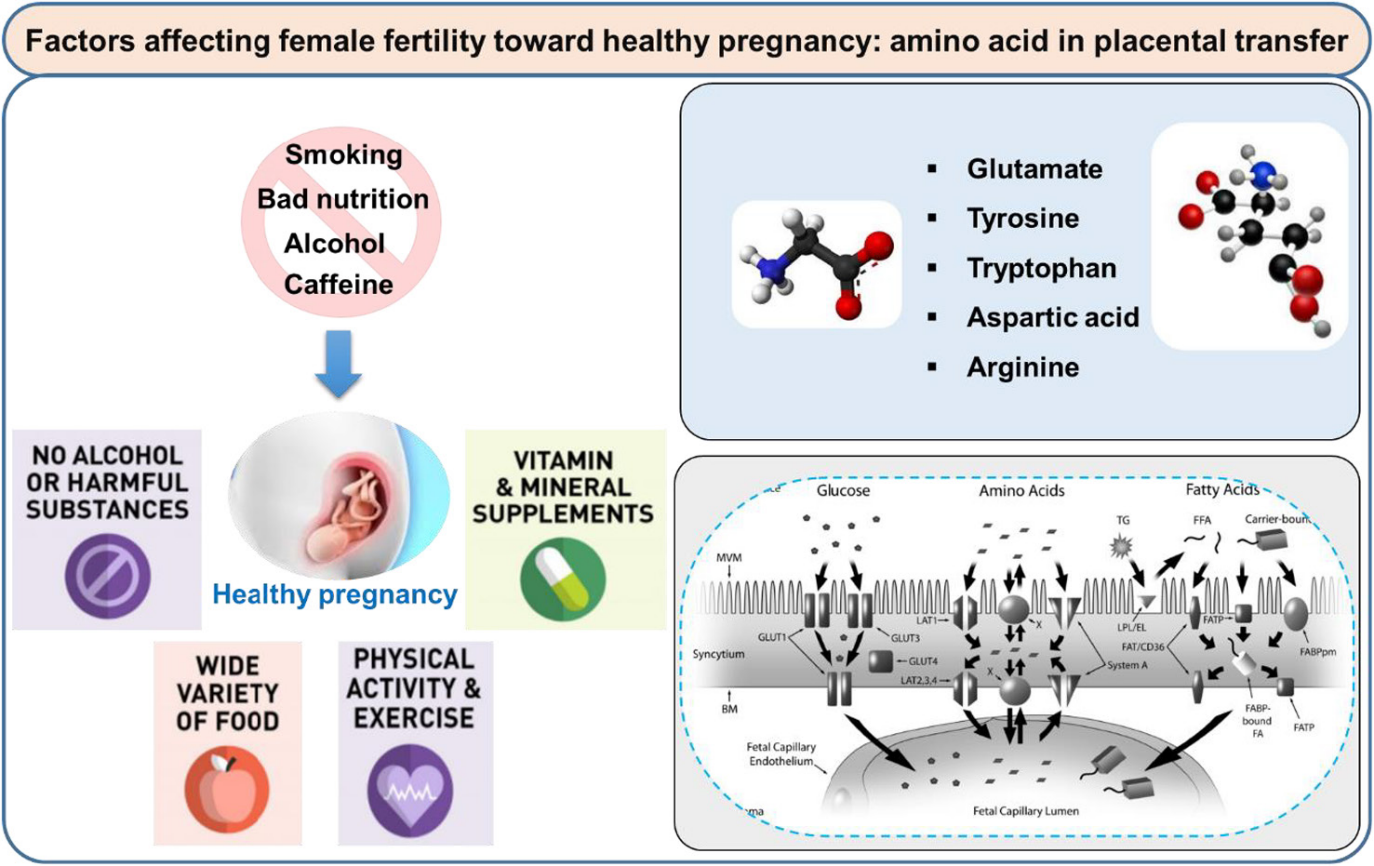
Factors affecting female fertility toward healthy pregnancy including good and bad habits. A specific illustration on functional amino acids (AAs) (glutamine, tyrosine, tryptophan, arginine, and aspartate) as essential nutrients and their transfer through placenta to the fetus

The relationship between different biological processes in living organisms depends mainly on nutrition. Food is vital for all creatures to extract energy and carry out all essential processes such as reproduction. Nutrients are essential for all the developmental stages in humans including growth, puberty, and reproduction (Ng et al., 2019). Reproduction is a process (involving sexual differentiation, maturation, gametogenesis, fertilization, and embryo development) that propagates a new life and ensures the continuation of the living species and the preservation of the offspring. It has been estimated that up to 15% of men and women are infertile worldwide, particularly in the industrialized nations (Sharma et al., 2013). Nutritional factors play a key role in determining the reproductive health and can positively or negatively influence fertility in humans. This topic is alarming, as validated by the great increase in the number of publications reported in the last 20 years (Figure 1). Therefore, it is necessary to know the appropriate type of food and the effect of each dietary material on the reproductive system, especially on the fertility of males and females (Figure 2).

The consumption of vegetable proteins rather than carbohydrates or animal proteins shows a substantially lower risk of ovulatory infertility (Chavarro et al., 2008). Carbohydrates are important for carrying out various biological processes and producing energy. Sugar intake is also an important factor that contributes to the daily caloric intake, and excessive sugar intake can lead to the development of chronic diseases such as obesity and type 2 diabetes (T2D), which have negative implications on fertility (Malik et al., 2010; Whitworth et al., 2011). However, dietary fatty acids and their unbalanced intake might impair metabolic homeostasis and fertility in premenopausal women, in quantitative and qualitative terms. Some micronutrients and food supplements, such as folic acid, calcium, zinc, selenium, antioxidants, iron, and vitamins D, B12, E, and C, have a potential effect on the health of the male and female reproductive systems, especially in pregnant women. Food supplements must be taken, but with caution because excessive intake can negatively affect the human health in general.

The omics approaches have been developed in many biological fields to identify potential biomarkers related to several diseases, including human infertility. Omics technologies elucidate how diet and definite nutrients interact with genes, proteins, and metabolites and affect metabolic phenotypes and disease consequences. It explains certain biological networks and nutrient detecting mechanisms attributed to metabolic variability. Therefore, omics approaches are significant in providing an enormous amount of information about biological processes involved in reproductive systems, and subsequently, raise the possibility of identifying biomarkers for the prediction and early diagnosis of diseases (Egea et al., 2014). Transport of amino acids (AAs) in the placenta is important during pregnancy because impaired placental amino acid transfer reduces fetal growth, which causes complications during the perinatal period and increases the susceptibility of acquiring chronic diseases (Cleal et al., 2018). The metabolomics approach explores the metabolism of dietary substrates that help in the treatment of chronic metabolic disorders (Gibney et al., 2005; Whitfield et al., 2004).

This review aims to clarify the importance of dietary substrates in the reproductive health in humans. Moreover, a full comprehension of different factors affecting human reproduction and fertility (including dietary interventions, lifestyle, physical activity, and genetic modifications) has been reported to avoid impairments and alterations in metabolic pathways. Recent advances in omics (including metabolomics and nutrigenomics) have addressed the effect of diet on the human metabolic regulation and human reproductive system.

## 2 Introduction

### 2.1 Nutrition, puberty, and reproduction in humans

The timing of puberty has an effect on public health, clinical, and social implications. Puberty encompasses a series of physical and psychosocial changes during the transition from childhood to young adulthood that prepare the human body for reproduction. Puberty is a potential biological occasion that needs maturation of reproductive neuroendocrine axis, episodic gonadotropin‐releasing hormone (GnRH), and luteinizing hormone (LH). Leptin plays a significant role in carrying metabolic information to the brain for puberty control. Since the GnRH neurons do not express the leptin receptor, the upstream neuronal network has to mediate the impact of leptin on GnRH secretion (Cardoso et al., 2020). Sex hormones are responsible for the physical manifestations of puberty. These include thelarche, which is the onset of breast development; pubarche, the appearance of pubic hair; gonadarche, the onset of sex hormone production by the gonads; menarche, the initiation of menses; and spermarche, the appearance of spermatozoa in semen (Villamor & Jansen, 2016). Nutrition is a significant factor affecting puberty neuroendocrine control. Though nutrient constraints during juvenile development cause postponements in puberty, high rates of body weight gain during this period enable pubertal maturation by programming hypothalamic centers that motivate the pubertal process. Intake of animal foods has been associated with an earlier onset of puberty in many surveys, involving a protein‐mediated enhancement of growth factor expression (Villamor & Jansen, 2016).

Obesity has a negative impact on the health of the male and female reproductive systems, and people who suffer from obesity always have low fertility rates. For this reason, obesity must be treated immediately (İrez et al., 2019). Recent studies propose that maternal nutrition throughout gestation can also control the development of the fetal neuroendocrine axis, thus persuading puberty and successive reproductive function. Intake of vegetable protein during childhood delays pubertal development (Santoro et al., 2019).

### 2.2 Effect of micronutrients and macronutrients on reproduction

The rate of human infertility is increasing continuously, which makes it a worldwide concern, and it contributes to approximately 50% of the problem in infertility cases. Male fertility potential is clinically examined by the semen analysis. There are a wide range of macronutrients and micronutrients, such as galactose, fructose, amino acids, zinc, potassium, magnesium, and vitamin C, which form the components of semen. The ability of semen to fertilize the female ovule is dependent on important factors including the quality and quantity of the sperm. Sexual reproduction in mammals is highly organized, which includes the generation of mature reproductive cells (oocyte and sperm) and the secretion of fluids (e.g., uterine secretions and seminal plasma) in the reproductive system of both males and females. Reproduction as a biological process includes several steps: the transference of sperm to the female reproductive tract, oocyte fertilization, zygote development, pregnancy recognition, embryo implantation, pregnancy maintenance, parturition, and lactation, as well as the growth and development of neonates (Bazer & Development, 2012; Owen & Katz, 2005). The above‐mentioned biological processes and several vital nutrients (minerals, carbohydrates, lipids, amino acids [AAs], and vitamins) are essential for the maturation of reproductive cells and the production of proteins, hormones, and secretions (Lin et al., 2014; Wu et al., 2012).

### 2.3 Micronutrients

**FIGURE 3:**
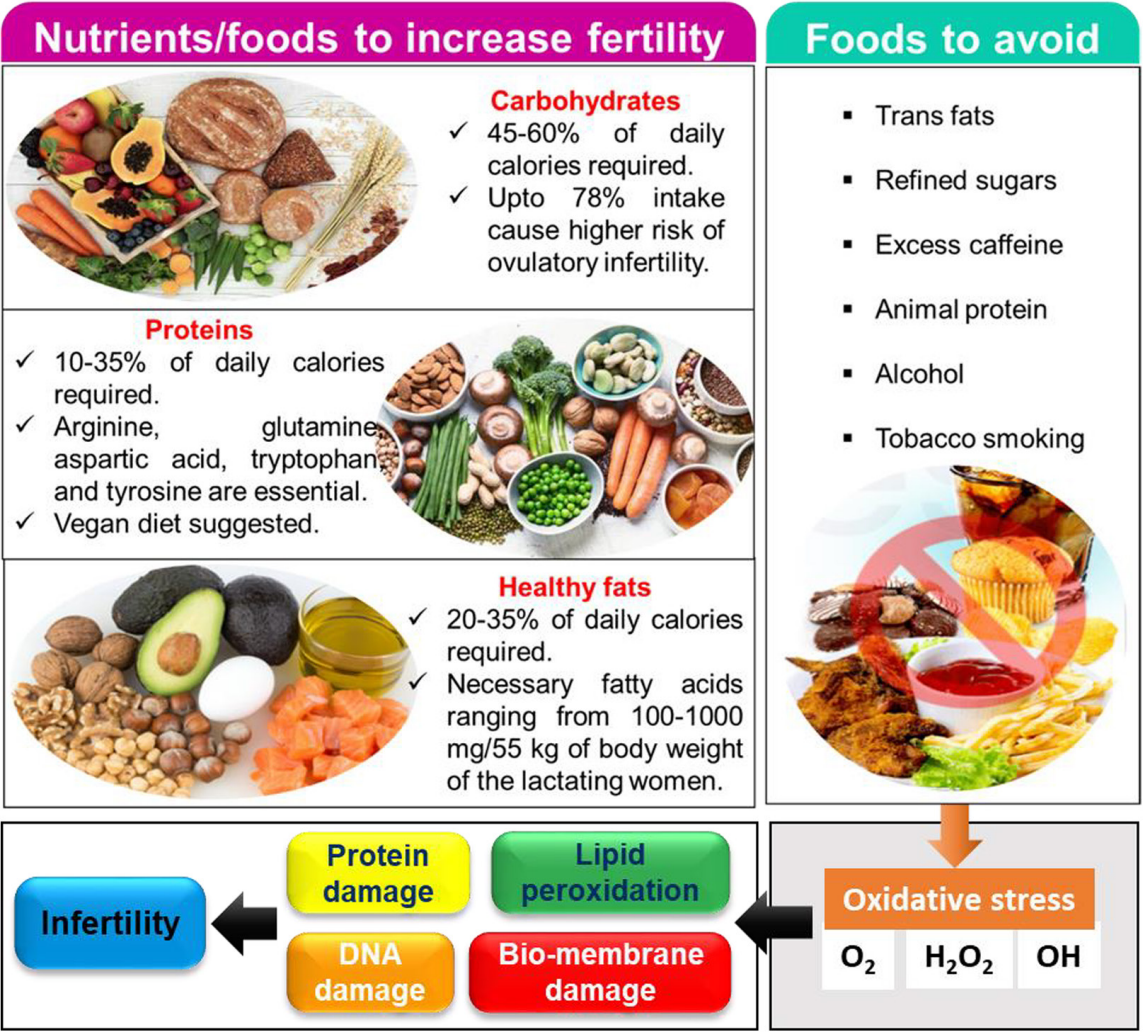
Essential foods (nutrients) to enhance the fertility in women. Carbohydrates, proteins, and healthy fats are macronutrients that are necessary for pregnant or lactating women. Few foods, which include animal proteins and transfats, should be avoided, as they cause infertility by different factors that induce production of reactive oxygen species

The dietary intake has a great impact on the health, development, and function of the human reproductive system, although the specific mechanisms have not been fully clarified yet. The genetic distinction that affects the metabolism of a nutrient may influence fertility by nutrigenetic mechanisms (Camus et al., 2020). Males and females require different nutrients for the expression of reproductive traits, as they have various roles in reproduction (Figure 3). Nevertheless, both sexes may require the regulation of nutrient intake, which maximizes sex‐specific fitness due to their shared genome (Ng et al., 2019). Isoflavones have a negative effect on the fertility of men; however, they assist the sexual health of menopausal women. Consumption of whole milk increases the fertility in women, but for men, the same benefit comes from the consumption of skimmed milk (Silva et al., 2019). Concerning dietary supplements, the role and performance of nine essential micronutrients are highlighted in Table 1.

**TABLE 1:** Description, role, and performance of essential micronutrients, and their impact on female and male reproductive health

Micronutrient	Description	Effect on female	Effect on male	Recommended dose	Consequences of deficiency	Sources	References
Folic acid	Known as vitamin B9 Essential compound involved in key biochemical processes	Improves chances of pregnancy Reduces risk of ovulatory infertility	Provides carbon for DNA synthesis and methylation Critical to spermatogenesis	400 μg/day	Premature birth Reduced intrauterine growth Increases risk of diabetes‐associated congenital disabilities	Vegetables, fruits, nuts, seafood, eggs, dairy, meat	Barchitta et al. (2020), González Rodríguez et al. (2018), National Institutes of Health (2008)
Calcium	Plays a role in reproductive health Facilitates fertilization	Creates alkaline environment in vagina Follicular production Oocyte activation and maturation	Regulates sperm motility	1 g/day	Hypertensive disorders of pregnancy Osteopenia, paranesthesia Muscle cramps, tetanus Delayed fetal growth Mineralization in the fetus	Dairy products, cabbage, kale, broccoli, almonds, tofu, sardines with bones	Peacock (2010), Simopoulos (1999)
Iron	Maintenance of healthy red blood cells Oxygen transport in the blood Immune function Free radical homeostasis	Helps the fertilized ovum implantation process	Essential to ejaculate fluidity Maintains sperm pH Sources of ferritin, which protects testicular tissue Developing sperm	30–60 mg/day	Risk of preterm birth Decreased defenses against infection Abnormal psychomotor development and cognitive impairment in infancy	Beans, vegetables, cereals, breads	Martin et al. (2016), Tremellen and Pearce (2015)
Vitamin B12	Known as cobalamin Cofactor in DNA and fatty acid synthesis Amino acid metabolism	Prevents spontaneous abortion Necessary for the development and functionality of the placenta	Improves the sperm quality	50 µg/day	Associated with abnormal estrogen level that interferes with implantation of fertilized egg	Fish, meat, poultry, eggs, milk	González Rodríguez et al. (2018), Visentin et al. (2016)
Selenium	Selenoprotein Plays a potential role in both female and male fertility	Placenta development Adequate development of the fetus’ nervous system	Maintains the spermatozoa integrity and viability Protects them from oxidative damage	60 µg/day	Increases risk of pregnancy complications Fetal growth restriction Increases thyroid hormone Alters the placental function	Nuts, seafood, fish, shrimp, muscle meats, cereals, dairy products	Mistry et al. (2012), Qazi et al. (2018)
Zinc	Plays a key role in fertility for both female and male Has a greater importance for men	Involved in capacitation and fertilization in the female reproductive tract	Testosterone synthesis Sperm viability Testicle development	20 mg/day	Preterm delivery Stillbirth Fetal neural tube defects Fetal growth restriction	Oysters, eggs, red meat, poultry, seafood, beans, nuts, grains, dairy	Kerns et al. (2018), Van Tienhoven (1968)
Vitamin E	A vital antioxidant in the cell membrane Supports reproductive functions	Participates in fertilized egg cell implantation and placenta development	Supports reproductive function in men Increases sperm quality and quantity	22–30 mg/day	Placental aging Vascular endothelial injury Disorders of pregnancy Placental abruption Abortion Premature birth	Nuts, seeds, vegetable oils, green leafy vegetables, fortified cereals	Buhling and Grajecki (2013), Rosen and Gallagher (2011)
Vitamin A	Supports the immune system Protects the gonads, reproductive tissues from oxidative stress	Affects ovarian follicular growth, uterine environments, and oocyte maturation	Has influence on sperm morphology and concentration	370 µg/day	Stops puberty in females and males Predisposes to low rates of fertilization and embryo mortality Reduces male sexual desire	Liver, fish oil, eggs, milk, leafy greens, vegetables, tomatoes, fruits	Cordova‐Izquierdo (2016), Simopoulos (1999)
Vitamin C	Aiding in tissue, hormone development Cofactor for enzymes, reducing oxidative damage	Essential for collagen biosynthesis Vital for adequate ovarian follicle growth and also for the ovulation and luteal phases	Affects the integrity and structure of sperm Promotes an environment for sperm to thrive	85 mg/day	Incidence of severe preeclampsia	Citrus, berries, pepper, kiwis, broccoli, brussels sprouts, tomatoes, potatoes	Buhling and Grajecki (2013), National Institutes of Health (2008)

Treatment and improvement of fertility in couples have been explored with a daily tablet from some certified products such as Fertility Support, FertiliWhey, and OvaBoost. The application of investigated nutrients in the form of commercial products would provide a complete source of proteins and all essential amino acids, support balanced blood sugar levels, and maintain healthy weight for natural reproductive support. They promote the quality, motility, volume, morphology, function, and count of sperm in males. As well, they improve polycystic ovary syndrome (PCOS), cycle, and/or hormone irregularities and support egg quality in females. Moreover, daily tablets of the patented blend comprising a mixture of Vitex agnus‐castus (Vitex) extract, active folate, and *Lepidium meyenii* (Maca) extract alone or with a gel capsule of minerals, vitamin, oligo‐elements plus docosahexaenoic acid (DHA) and eicosapentaenoic acid (EPA) omega 3‐fatty acids were formulated (ANTOINE et al., 2019). Such ingestible supplements and nutrients were applied to help and prepare the female body for conception and to improve women health during and around the time of pregnancy (Thierman & Hallaj, US20120040018 (2012)).

### 2.4 Macronutrients

#### 2.4.1 Carbohydrates

Sugar intake is an important factor, contributing to the daily caloric intake, and in excess, it can drive the development of chronic diseases such as obesity and type 2 diabetes (T2D) (Malik et al., 2010), which have negative implications on fertility (Whitworth et al., 2011). Intake of cookies and beverages sweetened with sugar had an opposite relationship with sperm progressive motility, with a risk of azoospermia (Alizadeh et al., 2017). In diabetics, a strong relationship between women's infertility and reduced insulin sensitivity was observed. The PCOS among women of reproductive age confirms that the quantity and quality of carbohydrates in diet would influence reproductive health functions (Fontana & Torre, 2016).

The nurses’ health study‐II (NHS‐II) found a relationship between the glycemic load and the risk of anovulation. A positive connection between total carbohydrate consumption and ovulatory infertility was also observed. When this macronutrient was taken in higher amounts, 78% higher risk of ovulatory infertility was observed (Chavarro et al., 2009). However, no significant relationship was found between total fiber intake and ovulatory infertility. Association between fiber‐rich diets and anovulation is not clear, as several studies have presented different effects (Chiu et al., 2018).

#### 2.4.2 Protein

As a nutrient, protein supplies amino acids, which are needed to carry out vital processes and provide energy. Each particular protein has a distinctive sequence of amino acids. There are 20 amino acids; ten of them (nonessential amino acids) can be synthesized sufficiently by the body to meet its demands, while the other 10 (essential amino acids) must be obtained from the diet (Hansen, 2016). Functional amino acids, such as arginine, glutamine, aspartic acid, sulfur‐containing AAs, tryptophan, and tyrosine, play a role in fertility and reproduction (Table 2). During pregnancy, certain AAs are necessary for specific processes (implantation, placental growth, angiogenesis, and the transfer of nutrients from the mother to the fetus) (Herring et al., 2018).

**TABLE 2:** Positive and negative impacts of various parameters on sperm and male fertility

Parameter	Positive impact	Negative impact	References
Obestatin	Increases testosterone secretions Ameliorates testicular functions	–	İrez et al. (2019)
Omega−6 fatty acids	Induces inflammation of a slight intensity, atherosclerosis, dysfunction of the endothelium, and oxidative stress	Cause a deterioration in testicular endocrine function Reduce the concentration levels of free and total testosterone Decrease the testicular volume	Mínguez‐Alarcón et al. (2017)
Saturated fats	–	Decrease the sperm concentration in semen Lower semen count	
Fish oil supplements	Improve semen parameters in response to omega‐3	Affect sperm fatty acid	
Grape seed Para–amino‐benzoic acid Red clover	Fertility‐enhancing dietary and nutritional supplement compositions	–	Andrews and Grunebaum (2015)
Amino acids (D‐Aspartic acid, arginine [Arg], glutamate, tyrosine, tryptophan)	Effective primary defense for protection of the sperm membrane structure Amino acids–deficient diet decreases sperm counts by ca. 90% and increases the percentage of nonmotile sperm approximately 10‐fold Amino acid supplements increased sperm counts by 18% and sperm motility by 7.6%	–	
Seminal plasma amino acids (Alanine, serine, valine, glycine, L‐proline, L‐glutamine)	Cause post‐thaw viability Enhance the live sperm, total motility, and maintain higher functional membrane and acrosomal integrity by reducing lipid peroxidation	–	Kocabaş et al. (2019)
Smoking	–	Induces oxidative stress in the testes Reduces sperm concentration Increases abnormal morphology Decreases motility and vitality Enhances DNA fragmentation and seminal leukocyte concentration Reduces capacitation and acrosome reactions	Martin et al. (2016)
Alcohol	–	Causes poor fertility Affects negatively spermatogenesis and semen parameters, including sperm motility, morphology, and concentration	Karmon et al. (2017)

High‐protein diets have been found to affect female fertility, as they delay the continuation of estrous cycles after calving, decrease fertility, and increase days from calving to conception (Fontana & Torre, 2016). Intake of protein sourced from animals has received attention in the context of fertility, mostly because of their potential to contain high levels of environmental contaminants, which could adversely affect reproductive health (Figure 3). Thus, it can be suggested that intake of protein from vegetal sources rather than animal sources provide a significantly lower risk of ovulatory infertility (Chavarro et al., 2008). The source and amount of protein in the diet have been reported to affect insulin sensitivity, which consequently stimulates ovulatory function (Layman et al., 2003). Two small trials have been performed to study the effects of a hypocaloric, low protein diet on reproductive function in overweight women with PCOS, compared to a high‐protein diet (Moran et al., 2003; Stamets et al., 2004). The protein content improved menstrual regularity and decreased circulating androgens with no effect on the reproductive function (Sørensen et al., 2012).

Replacement of carbohydrates with protein in diet improved glucose metabolism and weight loss, with no effect on sex hormone–binding globulin levels of testosterone (Tremellen & Pearce, 2015). Sufficient intake of dietary protein throughout pregnancy is vital for healthy pregnancy outcomes (King, 2000). Protein is essential not only for better development and growth of the fetus but also for maintenance of maternal tissues like blood, heart, uterus, breast, placenta, and fetal–support tissues of extra–embryonic membranes. Stephens et al. (2015) stated that the expected average necessity for protein in early gestation is 1.22 g/kg/day and that for late gestation is 1.52 g/kg/day. The common protein weight gain happens during the latter half of pregnancy, proposing their necessity for higher dietary protein till late gestation (Stephens et al., 2015). Nevertheless, a lot of the maternal adaptations concerning protein metabolism happens early in pregnancy, before there is an extensive increase in fetal need (Kalhan, 1998). The enlarged need for dietary protein noticed in early gestation shows that maternal adaptations to protein metabolism are recognized early in pregnancy. Balanced and optimal protein consumption is vital to prevent intrauterine growth limitation and infant low birth weight (Imdad & Bhutta, 2011). Thus, more understanding of maternal dietary protein necessities during pregnancy is essential to endorse fetal health.

Recently, there are several arguments about the impact of (1) animal protein, (2) dairy, and (3) soy products on fertility, as they have been connected with an improved intake of endocrine‐disrupting substances, pesticides, as well, growth factors and steroid hormones. All these aspects lead to variations in the hypothalamus–hypophysis–gonad axis and therefore the reproductive function (Chiu et al., 2018).

#### 2.4.3 Fats

Dietary fat has several important functions within the body. Its physiological roles include acting as an energy source, insulating organs, and playing a crucial part in the creation of hormones, cell membranes, and tissue membranes (Williams, 2000). Diets that contain high saturated fats lower sperm counts and overall sperm concentration. Spermatogenesis is negatively affected by trans‐fatty acids (TFAs) (Albert Salas‐Huetos et al., 2017). Polyunsaturated fatty acids (PUFAs) and TFAs are accumulated in the testes. The content and consumption of TFAs in semen causes poorer sperm quality; in addition, it lowers sperm concentration in the ejaculate. Moreover, studies on animals suggest that a trans‐fat‐rich diet reduces testosterone production and testicular mass, along with the initiation of pathological changes in the testes (Durairajanayagam, 2018; Veaute et al., 2007). High consumption of saturated fat was linked to low sperm count, while low‐fat milk intake is linked to increasing sperm count (Dattilo et al., 2014; Eslamian et al., 2016; Pant et al., 2015).

New nutritional compositions comprising essential fatty acids (EFAs) (linoleic acid, linolenic acid, arachidonic acid, docosahexaenoic acid, eicosapentaenoic acid, omega‐3 fatty acids, and omega‐6 fatty acids) were explored to improve neurological development of the embryo, fetus, and child, to provide nutritional support for women before and during lactation, and to improve gestational length and birth weight (Manning & Maggio, 2006). The necessary fatty acids should be ranging from 100 to 1000 mg/55 kg of body weight of the pregnant or lactating woman. Omega‐6 fatty acids affect fertility adversely, as they tend to increase inflammation of slight intensity, atherosclerosis, dysfunction of the endothelium, and oxidative stress (DiNicolantonio & O’Keefe, 2018). A study conducted on 209 healthy men showed that the consumption of omega‐6 fatty acids causes a deterioration of testicular endocrine function and decreases the testicular volume, as they reduce the concentration levels of free and total testosterone (Mínguez‐Alarcón et al., 2017). On the other hand, according to another study performed on 701 healthy men, the intake of saturated fats decreases the sperm concentration in semen and lowers the semen count (Jensen et al., 2013). In both studies, the lifestyle, diet, and health of the patients were considered (Mínguez‐Alarcón et al., 2017; Skoracka et al., 2020). Moreover, there is a negative correlation between the cholesterol level and semen volume (Pant et al., 2015). Fish oil supplementation in the diet of infertile male induced sperm fatty acids and improved parameters of semen in response to omega‐3 (Hajifoghaha et al., 2016).

In women, fats play a significant role in reproductive health, where the type of fats greatly affects fertility. Omega‐3 (n‐3) and PUFAs are essential for female fertility, as they are required for the production of substrates involved in implantation and pregnancy maintenance. They are also involved in oocyte maturation and embryo development. TFAs showed a negative effect on fertility by inducing insulin resistance, which made alterations in the ovary function and subsequently caused ovulatory infertility (Chiu et al., 2018; Gaskins & Chavarro, 2018). Therefore, fertility can be increased by the consumption of a diet rich in PUFAs and omega‐3 fatty acids and low in TFAs. However, the impact of saturated fatty acids (SFAs), monounsaturated fatty acids (MUFAs), and omega‐6 PUFAs on female fertility needs further investigation (Chiu et al., 2018). Overall, fast‐food products, ready‐made confectionery, sweet and salty snacks, and processed or red meat are the major sources of harmful fatty acids in the diet (De Souza et al., 2015).

## 3 THE CORRELATION BETWEEN AMINO ACIDS AND REPRODUCTION

There are 20 proteogenic amino acids that are the building blocks of protein synthesis (Canfield & Bradshaw, 2019). These are not only the building blocks of peptides and proteins but also are necessary for the production of many bioactive molecules that contribute to the regulation of metabolism and signaling pathways in the body (Dai et al., 2015). If too much protein is provided in the diet, only part of it will be consumed to create new proteins, and the remaining will be converted to energy (Wilson, 2003). Conversely, inadequate protein in the diet reduces growth and causes a loss of weight due to withdrawal of protein from less vital tissues to conserve the functions of more vital tissues. Thus, it is essential to explain the role of AAs in human reproduction and clarify the strong correlation between AA nutrition and reproduction (from spermatogenesis to oocyte fertilization and embryo implantation).

For instance, apolipoprotein E (ApoE) is a glycoprotein consisting of 299 amino acids and is highly produced in the ovaries. The main function of the ApoE is to transport cholesterol from the peripheral tissues to be metabolized in the liver. The carriage of ApoE4, one of the ApoE isoforms, during life span are being beneficial to women fertility and increased risk for developing cardiovascular and late‐onset Alzheimer's diseases (Oriá et al., 2020). Moreover, whether ApoE4 acts as an antagonistic pleiotropic allele, over the life span, affecting ovarian follicular growth and viability and overall women's reproductive system is still a matter of debate among many researchers. There is still a need for more studies to unravel the fine mechanisms modulating cell growth signaling pathways in the mammalian ovary under distinct environmental conditions and ApoE isoforms. Dietary and nutritional supplements for enhancing fertility have been investigated in male infertility therapy by providing para–amino‐benzoic acid (PABA), grape seeds, and red clover (Andrews & Grunebaum, 2015). Moreover, for female fertility therapy, a fertility‐enhancing composition was formulated with a dose of PABA, which supports optimal blood levels of folic acid, an interleukin‐6 (IL‐6) inhibiting dose of grape seed, and a dose of red clover comprising phytoestrogenic isoflavones. Dietary involvement with functional AAs, prebiotics, and probiotics to modify the activity of intestinal bacteria would improve reproductive performance in both males and females as well as their offspring (Bazer & Development, 2012). A continued supply of these nutrients helps the body to synthesize certain molecules that boost the reproductive system and support the user to achieve conception within a few months.

### 3.1 Functional amino acids in reproduction

The existence of AA metabolites and the maternal gut microbiota in the endometrium, placenta, and breast milk is likely a distinctive mark for the programming of the microbiome and metabolism in both the fetus and the infant body (Dai et al., 2015). AAs are metabolized by microbes in the small‐intestinal lumen, and therefore, their entry to the portal circulation for whole‐body utilization could be affected. Variations in their composition during pregnancy and plenty of AA metabolizing bacteria in the gut may change uterine function and epigenetic alterations of maternal metabolism and physiology.

Functional AAs are those AAs that control the metabolic pathways and improve health, growth, survival, development, lactation, and reproduction (Wu, 2013). Several studies showed that the seminal plasma is rich in threonine, serine, glutamate, glycine, tyrosine, and arginine, while allantoic fluid contains alanine, citrulline, glutamine, serine, arginine, glycine, and ornithine (Figure 2). Moreover, uterine secretions and milk are rich in leucine, arginine, proline, glutamine, aspartate, and taurine. Such abundance of AAs indicates their significant functions for human fertility, embryonic survival, as well as offspring development and growth (Table 2). Therefore, specific AA supplementation has valuable effects in improving reproductive performance for both humans and animals through signaling and metabolic pathways (Wang et al., 2012). Considering the contrary effects of a high‐protein diet on the health and metabolism of pregnant women and their offspring, dietary supplementation in an adequate amount can be beneficial for reproduction (Wu et al., 2013).

### 3.2 Amino acids required during pregnancy

Particular AAs are essential for specific processes involved in pregnancy such as placental growth, implantation, angiogenesis, and the transfer of the nutrients from the mother to the fetus (Figure 2). The disparity of AAs causes embryonic loss with impaired development and growth of the conceptus. Arginine (Arg) and glutamine (Glu) supplementation during exact phases of gestation helps overcome the destructive consequences of maternal protein malnutrition and helps improve embryonic survival and growth by enhancing placental angiogenesis and blood flow, as well as by stimulating embryonic protein synthesis. Moreover, leucine, Glu, Arg, and proline (Pro) play a significant role in the placental and fetal development (Martin et al., 2003; Wu et al., 2008). Protein requirement in maternal and fetal tissues enhances throughout pregnancy, mostly during the third trimester. Requirements for threonine improved by 55%, lysine by 45%, tryptophan by 35%, isoleucine by 63%, and during late stages of pregnancy when compared with those of the early stages (Elango & Ball, 2016).

### 3.3 Amino acid metabolism and transport in the placenta

Amino acid transmission in the placenta is a complex process that is vital for fetal development as it offers the necessary AAs for appropriate development and growth of the fetus (Cleal et al., 2018). The AA plasma concentrations are higher in the fetus than in the mother, reflecting the active transference of AAs through the human placenta (Jansson, 2001). The syncytiotrophoblast and the fetal capillary endothelium are the only two cell layers between the maternal and fetal circulation in the human placenta (Figure 2). Blood flow, surface area available for exchange, distance where diffusion occurs, and paracellular leak are important features for the efficiency of amino acid transport (Cleal et al., 2018).

Amino acid metabolism can affect placental amino acid transfer through its impact on both relative and total concentrations. In addition, it will influence arterial plasma AA concentrations and consequently the gradients that determine uptake and efflux. Placental protein synthesis will decrease the available AAs for transport, while the degradation of those proteins will increase the AA availability. Amino acid oxidation within the placenta is combined with forming a new AA such as alanine or glutamine through interconversion and prevention of toxic ammonia release. The synthesis of glutamine will create gradients that drive the uptake of other extracellular AAs (Day et al., 2013).

## 4 NUTRITION PATTERN AND A DIETARY MODEL SUPPORTING MALE FERTILITY

### 4.1 Role of AAs in sperm motility

Amino acids show an eminent role in multiple biologic and psychological processes and antioxidant properties that are an active primary defense for the protection of sperm membrane structure (Kocabaş et al., 2019). In the reproduction process, seminal plasma is a crucial biological fluid that controls sperm function (Table 2). Santiago‐Moreno et al. (2019) studied the total protein and AA profile of seminal plasma in 12 Spanish chicken breeds and explored the role of seminal plasma on the cryoresistance of rooster sperm. Glutamic acid was the most abundant free amino acid in seminal plasma, followed by alanine, serine, valine, and glycine. There was a positive relationship between seminal plasma concentrations of these AAs and post‐thaw viability. Fragmentation of DNA was minor in the absence of seminal plasma while the sperm viability was highly reduced (Figure 3). It is established that specific seminal plasma AAs were connected with post‐thaw percentage of DNA integrity and viable sperm. The exclusion of seminal plasma reduced the variability of the results and DNA fragmentation damages.

The addition of 25 mM of l‐proline and 20 mM of l‐glutamine enhanced the live sperm percentage, total motility percentage, and helped retain the acrosomal integrity and the higher functionality of the membrane by reducing lipid peroxidation (Sangeeta et al., 2015). Therefore, they can be employed as semen additives to freeze raw semen, as they prohibit cryoinjuries to sperm and enhance the prefreeze and post‐thaw semen characteristics. However, “l‐alanine” reduced the total motility percentage and fast progressive spermatozoa, and improved the immotile spermatozoa percentage. Cabrita et al. (2011) examined the effect of extra addition of numerous amino acid components on postthawed sperm viability, motility, and DNA integrity of gilthead seabream (*Sparus aurata*) and European seabass (*Dicentrarchus labrax*). Antioxidant supplementation (vitamins and amino acids) to *D. labrax* and *S. aurata* did not induce the parameters of motility or viability significantly. Taurine and hypotaurine significantly decreased both DNA fragmentation parameters in *S. aurata* sperm, protecting DNA against strand breaks. Overall, a species‐specific effect depends on the type of the antioxidants used.

l‐tryptophan was tested as an extender of Tigris scraper (*Capoeta umbla*) sperm during cryopreservation (Kutluyer et al., 2019). The sperm motility rate and duration were induced when the cryomedia was supplemented with l‐tryptophan. However, an increase in the concentration of l‐tryptophan in the extender caused a substantial decrease in the motility rate of Tigris scraper (*C. umbla*) sperm. The ultimate results were found at 1 mM l‐tryptophan; however, no motile sperm were observed when 2 mM concentration was used. Further studies related to long‐term storage and reproduction management need to be performed. Moreover, the production of reactive oxygen species by sperm was reduced by supplementation in vitro with antioxidants (Im Yun et al., 2013).

### 4.2 Mediterranean diet

The Mediterranean diet (MD) is the most studied dietary pattern worldwide. For more than almost 60 years, numerous studies have surveyed its association with human health, revealing its beneficial properties. The MD is considered as an essential diet for profertility. Particular diet includes large quantities of whole meal products, fruits and vegetables, olive oil, and nuts, which have great potential to enhance fertility in women and men (Table 3). There are numerous benefits of MD which have been reported, mainly due to its antioxidant, anti‐inflammatory, and lipid‐reducing effects (Salas‐Huetos et al., 2019). In fact, this diet is recommended as a preventive measure against cardiovascular diseases, type 2 diabetes (T2D), and neurodegenerative diseases. The consumption of MD helps to improve the quality of semen, but there is a need of further research in this area to determine whether it may contribute to a higher chance of positive pregnancy outcomes (Tosti et al., 2017).

**TABLE 3:** Mediterranean dietary products beneficial for fertility in women and men

Mediterranean dietary products	Active substances	Benefits for women and men	References
Fresh fish	Polyunsaturated fatty acid (PUFA), omega‐3 Fat‐soluble vitamins A, D, E, and K	Women: lower the risk of obesity Men: sources of docosahexaenoic acid (DHA) and eicosapentaenoic acid (EPA) in the diet and associated with improvement in the quality of semen	Afeiche et al. (2014)
Eggs	Folate and B6	Women: increase both progesterone and estrogen levels, which regulate menstrual cycles and ovulation Men: support both the semen quality and semen count. They can also increase their levels of testosterone, which boosts libido	Salas‐Huetos, James et al. (2019)
Vegetables and fruit	Antioxidants, folic acid, fiber, minerals	Women and men: vegetables and fruits provide the basis for prohealthy nutrition models, which are associated with the improvement of semen quality and fertility	Ricci et al. (2018)
Nuts, seeds	Essential fatty acids (EFAs), fiber, tocopherols, phytosterols, polyphenols, minerals	Women: rich source of protein, minerals, and fatty acids which help to improve ovulation Men: it is important to choose nuts and unroasted and unsalted seeds. The use of nuts in the diet may have a beneficial effect on the quality of sperm	Salas‐Huetos et al. (2018)
Whole‐grain products	Fiber, zinc, magnesium	Women: increase the thickness of endometrial lining, which supports the implantation of an embryo Men: improve semen quality	Salas‐Huetos et al. (2017)
Lean dairy	Calcium, a wholesome protein	Women and men: dairy contains protein and other nutrients known to support fertility like zinc, choline, selenium, vitamin A, and vitamin D. Vitamin A is essential for reproduction in both.	Salas‐Huetos, James, et al. (2019)
Olive oil, rapeseed oil	PUFA, alpha‐linolenic acid, vitamin E, polyphenols	Women: improve the structure of reproductive cells Men: increase the level of testosterone and enhance fertility	Giahi et al. (2016)

### 4.3 Antioxidants for male fertility

Male infertility is a major clinical challenge, which is increasing rapidly, while male factors such as poor quality of semen are responsible for 25% of all infertility issues. Although oxidative stress tends to be the primary factor underlying male infertility, it should be stressed that studies on the efficiency of antioxidant therapy are still contradictory. In a study, it is reported that oral antioxidant supplementation improves the parameters evaluating semen quality and is associated with less DNA damage (Martin‐Hidalgo et al., 2019). The most frequently used antioxidants, both in monotherapy and combined supplementation, include vitamins E and C, carnitine, coenzyme Q10, zinc, selenium, folic acid, and N‐acetylcysteine (Showell et al., 2011).

Antioxidant supplementation has a remarkable benefit for male patients who have low sperm parameters. Wirleitner et al. (2012) observed a significant reduction in the percentage of immotile sperm cells with a drastic improvement in sperm motility and count. Considering the putative relationship between semen quality and reactive oxygen species, the changes in the sperm parameters indicate that a decline in semen quality, and even subtle morphological changes, might be associated with oxidative stress.

## 5 THE HAZARDOUS IMPACTS OF TOBACCO SMOKING, AND ALCOHOL AND CAFFEINE CONSUMPTION ON FERTILITY

Exposure to several environmental agents and lifestyle factors negatively affects reproductive health including, poor nutritional intake, obesity, smoking, recreational drugs (e.g., cannabis, opioids, cocaine, and anabolic steroids), and alcohol use. Intakes of caffeine and alcohol are, unquestionably, the most studied dietary factors as the potential disruptors of fertility.

### 5.1 Tobacco smoking

Cigarette smoke represents a well‐established combination of reproductive toxins for both women and men. Despite the association between tobacco use and the harmful effects on general health as well as fertility parameters, smoking remains globally prevalent. The usage of tobacco has increased rapidly among young girls aged 13–15 years all over the world (Watson, 2015). The main content of tobacco smoke is nicotine and its metabolite, cotinine, which causes extensive harm to germ cells. Early‐age smoking causes infertility, menopause, premature ovarian failure, and spontaneous abortion. Moreover, smoking during pregnancy causes the thickening of the villous membrane and reduction in the absorption of nutrients that diffuse through the placenta. Therefore, pregnant smokers have a high nutritional risk due to poor diet and lower levels of vitamins (Gustavson et al., 2017). Smoking induces oxidative stress in the testes, affects spermatogenesis and steroidogenesis, as well, and it has negative effects on sperm as it (1) reduces sperm concentration, (2) increases abnormal morphology, (3) decreases motility and vitality, (4) enhances DNA fragmentation and seminal leukocyte concentration, (5) reduces capacitation and acrosome reactions, (6) causes abnormal protein expression, and (7) gives rise to both genetic and epigenetic aberrations in spermatozoa (Martin et al., 2016).

In conclusion, not only the content of tobacco smoke is concerned in the pathogenesis of human infertility, but also its contents have been shown to be a mutagen and an aneugen of spermatozoa. Moreover, the genetic mutations are not only seen in the spermatogonial stem cells of the smoker, but also seen in the offspring.

### 5.2 Alcohol

Consumption of too much alcohol has detrimental influence on human health, including high risk of many cancers, heart failure, stroke, and death (Voelker, 2013; Wood et al., 2018). During pregnancy, alcohol consumption has negative effects on multiple fetal organ systems, and it causes poor fertility in both women and men, substantial complications in pregnancy, and adverse fetal development (Tan et al., 2015). Alcohol's toxicity reduces semen quality and causes impaired hypothalamic–pituitary–testicular axis (Sadeu et al., 2010). Moreover, it decreases female fertility with possible effects on the hypothalamus, which lowers luteinizing hormone secretion and anovulation. Such alcohol's teratogenic effects (especially on the embryo and fetus development), have encouraged general advisements for women to avoid drinking alcohol (Anderson et al., 2010). The consumption of moderate or acute (<5 units/week) amounts of alcohol appears to have a minimal effect on sperm parameters, improves assisted reproductive technique (ART) outcomes, and increase testosterone (Jensen et al., 2014). However, heavy and chronic (>20–25 units/week) consumption of alcohol affects spermatogenesis and semen parameters negatively, including sperm motility, morphology, and concentration (Karmon et al., 2017). Thus, irregular drinking of alcohol seems not to have an undesirable effect on the quality of semen; yet, regular consumption results in the worsening of both sperm morphology and semen volume.

### 5.3 Caffeine

Caffeine exists in various natural substances, including tea, coffee, chocolate, energy drinks, and cola‐containing soft drinks (Jensen et al., 2010). Caffeine intake still has an inconclusive effect on human reproduction. Some studies found that caffeine consumption has no potential effect on male fertility parameters (Karmon et al., 2017). However, more than 6 cups/day of coffee has been suggested to decrease fertility in couples (Hassan et al., 2004). Oluwole et al. (2016) concluded that consumption of 300 mg of caffeine per day is safe (Homan et al., 2007; Sharma et al., 2013). The impact of caffeine intake on prolonged time of pregnancy may be confused by other bad lifestyle habits such as smoking. Caffeine intake (<200–300 mg/day) may cause harmful reproductive consequences such as spontaneous abortion. Overall, it has been advised that women who are pregnant or trying to conceive should limit their caffeine intake to 100–200 mg/day.

## 6 NUTRIENT UPTAKE (NUTRIGENOMICS) AND METABOLISM (METABOLOMICS)

**FIGURE 4:**
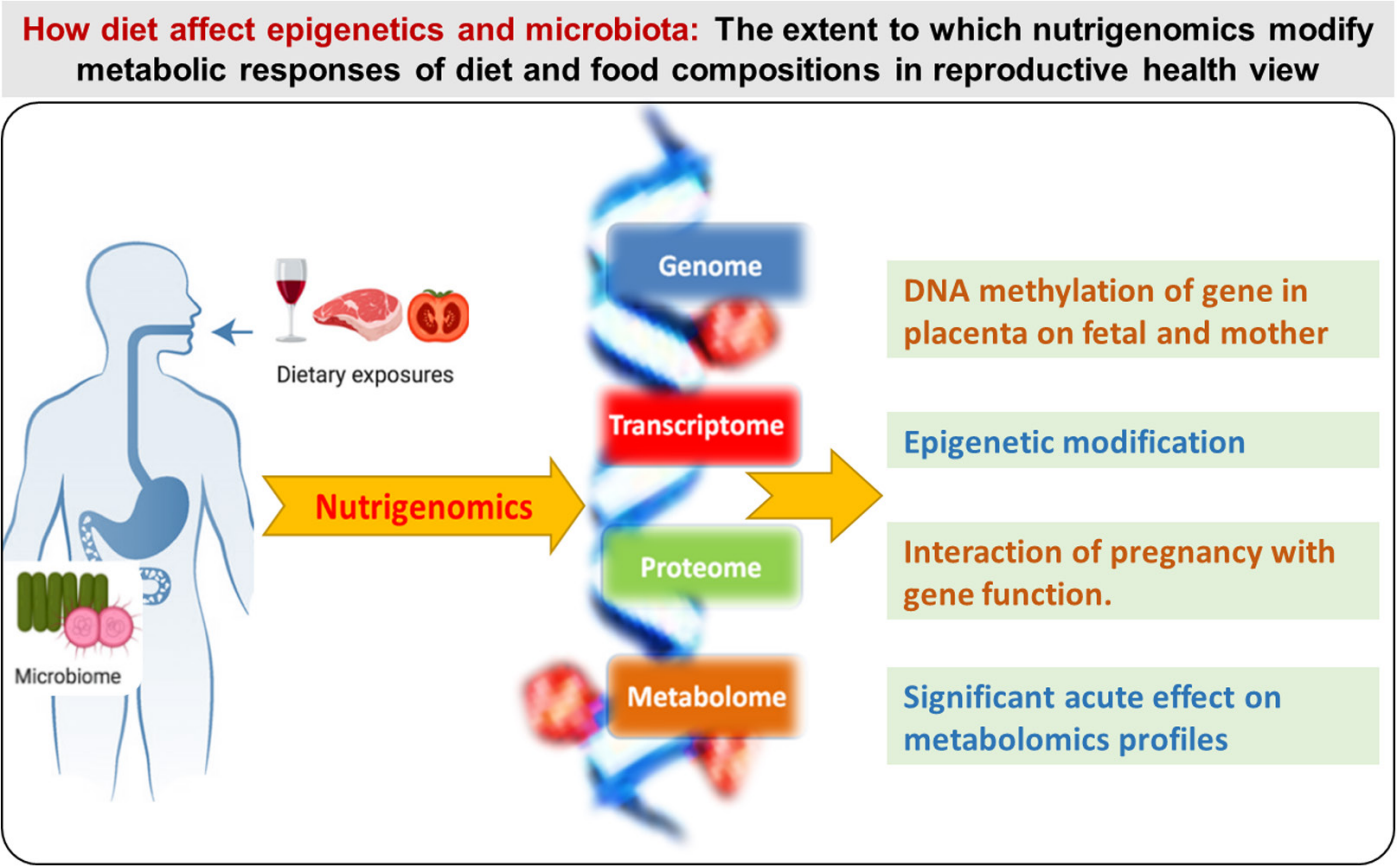
Effects of nutrition (nutrigenomics) and their metabolism (metabolomics) on genome, transcriptome, proteome, and metabolome of human reproduction (especially women and infant). Such high‐throughput technologies (omics approach) offer the opportunity to understand the flow of information that underlies the disease

Variations in the nutrient composition of the human diet cause changes in the metabolic profiles of individuals (Whitfield et al., 2004). Therefore, to recognize the effects of exogenous compounds on human metabolic regulation, receptiveness research on nutrigenomics and nutrigenetics should be performed (Gibney et al., 2005). The post–genomic era supported researchers to explore the nutrients’ effects on physiological functions at the molecular level. Metabolomics play a significant role in nutritional sciences with a high potential value, which has been demonstrated in several studies examining the metabolism of dietary compounds (Table 4). Metabolomics classifies metabolites related to fetal growth restriction (FGR) by investigating early and late pregnancy differences in urinary metabolites (Clinton et al., 2020). Moreover, novel molecules and pathways were identified in pregnant versus nonpregnant women (Handelman et al., 2019). Overall, high‐throughput technologies (omics approach) offer the opportunity to understand the flow of information that underlies diseases (Figure 4). However, huge challenges still remain, despite the substantial advances in this field.

**TABLE 4:** Omics approach and molecular techniques used to monitor the effect of diets on human reproduction

Diet	Omics approach	Molecular techniques used	Study goals	Summary	References
Gestational diabetes mellitus (GDM)	Metabolomics	DNA methylation Histone modification Alterations to noncoding RNAs	Explores the epigenetic modifications in fetal tissue which play a mechanistic role in metabolic disease	Maternal GDM influences future risk of obesity, impaired glucose tolerance (IGT), type 2 diabetes (T2D), and cardiovascular disease through the interaction of the pregnancy with gene function	Moholdt and Hawley (2020)
Ethyl glucoside	Metabolomics	^1^H nuclear magnetic resonance (NMR) spectroscopy	Investigates the role of dietary components	Dietary components showed a significant role in health and disease	Teague et al. (2004)
Isoflavones	Metabolomics	Biofluid ^1^H nuclear magnetic resonance (NMR)‐based			Solanky et al. (2003)
Two‐hour glucose concentrations	Metabolomics	DNA methylation of the leptin gene	Find significant correlations between 2‐h glucose concentrations and the degree of DNA methylation of the leptin gene in placenta on both the fetus and mother	Higher glucose values correlated with a lower magnitude of methylation on the fetus, but with a higher degree of methylation on the maternal side	Bouchard et al. (2010)
Gestational diabetes mellitus	Transcriptomics & Metabolomics	Expression of plasma microRNAs (miRNAs)	Detect the differential expression of 32 miRNAs whose targets were associated with insulin resistance and poor pregnancy outcomes (preeclampsia, emergency Cesarean section, and neonatal hypoglycemia)	Involved in mitochondrial function and glucose metabolism	Zhu et al. (2015)
GDM or GDM controlled by medication	Transcriptomics & Metabolomics	Transcriptional coactivator peroxisome proliferator–activated receptor gamma coactivator 1 alpha (PGC1‐α)	Detect the differential expression of miRNAs whose targets involved mitochondrial function and glucose metabolism	Lower protein levels were observed in both GDM groups compared with body mass index (BMI)‐matched controls	Muralimanoharan et al. (2016)
Salicyluric and salicylic acids (fruit and vegetables)	Nutrigenomics & Metabolomics	NMR and mass spectrometry (MS) technologies (cytochrome P450 1A2 pathway)	Study the potential of nutrients from plant foods to exert a significant acute effect on metabolomic profiles, especially the changes in urine	Genotype interaction was observed	Lawrence et al. (2003)
Allylmer apturic acid (in garlic)					de Rooij et al. (1996)
GDM	Metabolomics	Metagenomics of gut microbiota	Examine the gut microbiota composition to be compared with that in normoglycemic pregnant women	The composition of the gut microbiota from pregnant women with GDM resembled the aberrant microbiota composition reported in nonpregnant individuals with T2D	Crusell et al. (2018)

## 7 CURRENT AND FUTURE DEVELOPMENTS

The value of nutrient supplementation in reproductive health is considerable. Some of the functional amino acids and fatty acids have shown their impact on improving female and male fertility. Moreover, a combination of micronutrients has been optimized and developed to be used as a tablet, which was convenient for use for pregnant women. Many patents disclosed effective supplementation to improve and treat infertility. It might be better to change them into products by cooperating with or transferring to companies. Despite all these encouraging remarks, the available clinical data are still inadequate and many important questions are still to be addressed about the role of amino acids in reproductive health. Longitudinal studies focused on the reproductive outcomes of diet to evaluate the differences in lifestyle, physical activity, and genetic variation need more attention. Also, a complete understating of changes in metabolic profiles (metabolomics) as a result of dietary exposure is required. These data open new windows of therapeutic intervention for the treatment of infertility in male and female patients.

## 8 CONCLUSIONS

The literature on the relationship between diet and human fertility has greatly expanded over the last decade, resulting in the identification of a few clear patterns. Recent advances in “omics” approaches provide the opportunity to search for fertility biomarkers, which could be used for predicting the fertility potential of young males prior to sexual maturity or to screen existing males for artificial insemination programs. Integration of metabolomics and nutrigenomics with reproductive health is very significant to identify the indicators of multifactorial and complex processes related to the physiology of human fertility. Inclusive, optimized nutrition preserves human health and fertility and represents the most attractive challenge that we have to take up. Future efforts should focus on jointly considering female and male diets. Furthermore, to overcome the limitations inherent to observational research based on nutritional biomarkers, it is essential that the most consistent associations are tested in adequately powered randomized controlled trials.

## CONFLICT OF INTEREST

The authors declare that they have no conflict of interest.

## AUTHOR CONTRIBUTION

**Xiaoling Ma:** Conceptualization (supporting); Investigation (equal); Project administration (lead); Supervision (equal); Visualization (equal). **Luming Wu:** Conceptualization (supporting); Data curation (equal); Investigation (supporting); Writing – original draft (equal); Writing – review & editing (equal). **Yinxue Wang:** Investigation (equal); Validation (equal); Visualization (equal); Writing – review & editing (equal). **Shiqiang Han:** Data curation (equal); Investigation (equal); Validation (equal). **Marwa M. El‐Dalatony:** Conceptualization (equal); Validation (equal); Writing – original draft (lead); Writing – review & editing (lead). **Fei Feng:** Funding acquisition (equal); Methodology (equal); Resources (equal). **Zhongbin Tao:** Formal analysis (equal); Investigation (equal); Resources (equal). **Liulin Yu:** Funding acquisition (equal); Software (equal); Visualization (equal). **Yiqing Wang:** Funding acquisition (equal); Project administration (equal); Supervision (equal).

## ETHICAL APPROVAL STATEMENT

This study does not involve any human or animal testing.

## INFORMED CONSENT

Informed consent was obtained from all study participants.

## Data Availability

Data sharing is not applicable to this article as no new data were created or analysed in this article.

## References

[fsn32708-bib-0001] Afeiche, M. C. , Gaskins, A. J. , Williams, P. L. , Toth, T. L. , Wright, D. L. , Tanrikut, C. , Hauser, R. , & Chavarro, J. E. (2014). Processed meat intake is unfavorably and fish intake favorably associated with semen quality indicators among men attending a fertility clinic. Journal of Nutrition, 144(7), 1091–1098. 10.3945/jn.113.190173 PMC4056648 24850626

[fsn32708-bib-0002] Alizadeh, S. , Mirmiran, P. , & Hajifoghaha, M. (2017). Role of nutrition in female and male fertility. Journal of Babol University of Medical Sciences, 19(4), 7–15.

[fsn32708-bib-0003] Anderson, K. , Nisenblat, V. , & Norman, R. (2010). Lifestyle factors in people seeking infertility treatment–a review. Australian and New Zealand Journal of Obstetrics and Gynaecology, 50(1), 8–20. 10.1111/j.1479-828X.2009.01119.x 20218991

[fsn32708-bib-0004] Andrews, K. & Grunebaum, A. N. (2015). Composition and method for fertility therapy using nutritional supplements Google patent US8974838.

[fsn32708-bib-0005] Antoine, E. , Chirila, S. , & Teodorescu, C. (2019). A patented blend consisting of a combination of *Vitex agnus‐castus* extract, *Lepidium meyenii* (Maca) extract and active folate, a nutritional supplement for improving fertility in women. Mædica, 14(3), 274. 10.26574/maedica.2019.14.3.274 PMC6861720 31798745

[fsn32708-bib-0006] Barchitta, M. , Maugeri, A. , Magnano San Lio, R. , Favara, G. , La Mastra, C. , La Rosa, M. C. , & Agodi, A. (2020). Dietary folate intake and folic acid supplements among pregnant women from Southern Italy: Evidence from the “Mamma & Bambino” Cohort. International Journal of Environmental Research and Public Health, 17(2), 638. 10.3390/ijerph17020638 PMC7013905 31963813

[fsn32708-bib-0007] Bazer, F. W. , & Development (2012). Contributions of an animal scientist to understanding the biology of the uterus and pregnancy. Reproduction, Fertility and Development, 25(1), 129–147. 10.1071/RD12266 23244834

[fsn32708-bib-0008] Bouchard, L. , Thibault, S. , Guay, S.‐P. , Santure, M. , Monpetit, A. , St‐Pierre, J. , Perron, P. , & Brisson, D. (2010). Leptin gene epigenetic adaptation to impaired glucose metabolism during pregnancy. Diabetes Care, 33(11), 2436–2441. 10.2337/dc10-1024 20724651 PMC2963508

[fsn32708-bib-0009] Buhling, K. J. , & Grajecki, D. (2013). The effect of micronutrient supplements on female fertility. Current Opinion in Obstetrics & Gynecology, 25(3), 173–180. 10.1097/GCO.0b013e3283609138 23571830

[fsn32708-bib-0010] Cabrita, E. , Ma, S. , Diogo, P. , Martínez‐Páramo, S. , Sarasquete, C. , & Dinis, M. (2011). The influence of certain aminoacids and vitamins on post‐thaw fish sperm motility, viability and DNA fragmentation. Animal Reproduction Science, 125(1–4), 189–195. 10.1016/j.anireprosci.2011.03.003 21482049

[fsn32708-bib-0011] Camus, M. , Moore, J. , & Reuter, M. (2020). Nutritional geometry of mitochondrial genetic effects on male fertility. Biology Letters, 16(2), 20190891. 10.1098/rsbl.2019.0891 32097597 PMC7058949

[fsn32708-bib-0012] Canfield, C.‐A. , & Bradshaw, P. C. (2019). Amino acids in the regulation of aging and aging‐related diseases. Translational Medicine of Aging, 3, 70–89. 10.1016/j.tma.2019.09.001

[fsn32708-bib-0013] Cardoso, R. , West, S. , Maia, T. , Alves, B. , & Williams, G. (2020). Nutritional control of puberty in the bovine female: Prenatal and early postnatal regulation of the neuroendocrine system. Domestic Animal Endocrinology, 73, 106434. 10.1016/j.domaniend.2020.106434 32115309

[fsn32708-bib-0014] Chavarro, J. E. , Rich‐Edwards, J. W. , Rosner, B. A. , & Willett, W. C. (2008). Protein intake and ovulatory infertility. American Journal of Obstetrics and Gynecology, 198(2), 210.e211–210.e217. 10.1016/j.ajog.2007.06.057 18226626 PMC3066040

[fsn32708-bib-0015] Chavarro, J. E. , Rich‐Edwards, J. W. , Rosner, B. A. , & Willett, W. C. (2009). A prospective study of dietary carbohydrate quantity and quality in relation to risk of ovulatory infertility. European Journal of Clinical Nutrition, 63(1), 78–86. 10.1038/sj.ejcn.1602904 17882137 PMC3066074

[fsn32708-bib-0016] Chiu, Y.‐H. , Chavarro, J. E. , & Souter, I. (2018). Diet and female fertility: Doctor, what should I eat? Fertility and Sterility, 110(4), 560–569. 10.1016/j.fertnstert.2018.05.027 30196938

[fsn32708-bib-0017] Cleal, J. K. , Lofthouse, E. M. , Sengers, B. G. , & Lewis, R. M. (2018). A systems perspective on placental amino acid transport. The Journal of Physiology, 596(23), 5511–5522. 10.1113/JP274883 29984402 PMC6265537

[fsn32708-bib-0018] Clinton, C. M. , Bain, J. R. , Muehlbauer, M. J. , Li, Y. Y. , Li, L. , O’Neal, S. K. , Hughes, B. L. , Cantonwine, D. E. , Mcelrath, T. F. , & Ferguson, K. K. (2020). Non‐targeted urinary metabolomics in pregnancy and associations with fetal growth restriction. Scientific Reports, 10(1), 1–8. 10.1038/s41598-020-62131-7 32210262 PMC7093500

[fsn32708-bib-0019] Cordova‐Izquierdo, A. (2016). Best Practices in animal reproduction: Impact of nutrition on reproductive performance livestock. Advances in Dairy Research, 4, 152.

[fsn32708-bib-0020] Crusell, M. K. W. , Hansen, T. H. , Nielsen, T. , Allin, K. H. , Rühlemann, M. C. , Damm, P. , Vestergaard, H. , Rørbye, C. , Jørgensen, N. R. , Christiansen, O. B. , Heinsen, F.‐A. , Franke, A. , Hansen, T. , Lauenborg, J. , & Pedersen, O. (2018). Gestational diabetes is associated with change in the gut microbiota composition in third trimester of pregnancy and postpartum. Microbiome, 6(1), 1–19. 10.1186/s40168-018-0472-x 29764499 PMC5952429

[fsn32708-bib-0021] Dai, Z. , Wu, Z. , Hang, S. , Zhu, W. , & Wu, G. (2015). Amino acid metabolism in intestinal bacteria and its potential implications for mammalian reproduction. Molecular Human Reproduction, 21(5), 389–409. 10.1093/molehr/gav003 25609213

[fsn32708-bib-0022] Dattilo, M. , Cornet, D. , Amar, E. , Cohen, M. , & Menezo, Y. (2014). The importance of the one carbon cycle nutritional support in human male fertility: A preliminary clinical report. Reproductive Biology and Endocrinology, 12(1), 1–9. 10.1186/1477-7827-12-71 25073983 PMC4119238

[fsn32708-bib-0023] Day, P. , Cleal, J. , Lofthouse, E. , Goss, V. , Koster, G. , Postle, A. , Jackson, J. , Hanson, M. , Jackson, A. , & Lewis, R. (2013). Partitioning of glutamine synthesised by the isolated perfused human placenta between the maternal and fetal circulations. Placenta, 34(12), 1223–1231. 10.1016/j.placenta.2013.10.003 24183194 PMC3851744

[fsn32708-bib-0024] de Rooij, B. M. , Boogaard, P. J. , Rijksen, D. A. , Commandeur, J. N. , & Vermeulen, N. P. (1996). Urinary excretion of N‐acetyl‐S‐allyl‐L‐cysteine upon garlic consumption by human volunteers. Archives of Toxicology, 70(10), 635–639. 10.1007/s002040050322 8870956

[fsn32708-bib-0025] de Souza, R. J. , Mente, A. , Maroleanu, A. , Cozma, A. I. , Ha, V. , Kishibe, T. , Uleryk, E. , Budylowski, P. , Schünemann, H. , Beyene, J. , & Anand, S. S. (2015). Intake of saturated and trans unsaturated fatty acids and risk of all cause mortality, cardiovascular disease, and type 2 diabetes: Systematic review and meta‐analysis of observational studies. BMJ, 351, h3978. 10.1136/bmj.h3978 26268692 PMC4532752

[fsn32708-bib-0026] DiNicolantonio, J. J. , & O’Keefe, J. H. (2018). Importance of maintaining a low omega–6/omega–3 ratio for reducing inflammation. Open Heart, 5(2), e000946. 10.1136/openhrt-2018-000946 30564378 PMC6269634

[fsn32708-bib-0027] Durairajanayagam, D. (2018). Lifestyle causes of male infertility. Arab Journal of Urology, 16(1), 10–20. 10.1016/j.aju.2017.12.004 29713532 PMC5922227

[fsn32708-bib-0028] Egea, R. R. , Puchalt, N. G. , Escrivá, M. M. , & Varghese, A. C. (2014). OMICS: Current and future perspectives in reproductive medicine and technology. Journal of Human Reproductive Sciences, 7(2), 73. 10.4103/0974-1208.138857 25191020 PMC4150148

[fsn32708-bib-0029] Elango, R. , & Ball, R. O. (2016). Protein and amino acid requirements during pregnancy. Advances in Nutrition: An International Review Journal, 7(4), 839S–844S. 10.3945/an.115.011817 PMC4942872 27422521

[fsn32708-bib-0030] Eslamian, G. , Amirjannati, N. , Rashidkhani, B. , Sadeghi, M.‐R. , Baghestani, A.‐R. , & Hekmatdoost, A. (2016). Adherence to the Western pattern is potentially an unfavorable indicator of asthenozoospermia risk: A case‐control study. Journal of the American College of Nutrition, 35(1), 50–58. 10.1080/07315724.2014.936983 25764357

[fsn32708-bib-0031] Fontana, R. , & Torre, S. D. (2016). The deep correlation between energy metabolism and reproduction: A view on the effects of nutrition for women fertility. Nutrients, 8(2), 87. 10.3390/nu8020087 26875986 PMC4772050

[fsn32708-bib-0032] Gaskins, A. J. , & Chavarro, J. E. (2018). Diet and fertility: A review. American Journal of Obstetrics and Gynecology, 218(4), 379–389. 10.1016/j.ajog.2017.08.010 28844822 PMC5826784

[fsn32708-bib-0033] Giahi, L. , Mohammadmoradi, S. , Javidan, A. , & Sadeghi, M. R. (2016). Nutritional modifications in male infertility: A systematic review covering 2 decades. Nutrition Reviews, 74(2), 118–130. 10.1093/nutrit/nuv059 26705308 PMC4892303

[fsn32708-bib-0034] Gibney, M. J. , Walsh, M. , Brennan, L. , Roche, H. M. , German, B. , & Van Ommen, B. (2005). Metabolomics in human nutrition: Opportunities and challenges. The American Journal of Clinical Nutrition, 82(3), 497–503. 10.1093/ajcn/82.3.497 16155259

[fsn32708-bib-0035] González Rodríguez, L. G. , López Sobaler, A. M. , Perea Sánchez, J. M. , & Ortega, R. M. (2018). Nutrición y fertilidad. Nutrición Hospitalaria, 35(SPE6), 7–10. 10.20960/nh.2279 30351153

[fsn32708-bib-0036] Gustavson, K. , Ystrom, E. , Stoltenberg, C. , Susser, E. , Surén, P. , Magnus, P. , Knudsen, G. P. , Smith, G. D. , Langley, K. , Rutter, M. , Aase, H. , & Reichborn‐Kjennerud, T. (2017). Smoking in pregnancy and child ADHD. Pediatrics, 139(2), e20162509. 10.1542/peds.2016-2509 28138005 PMC5260151

[fsn32708-bib-0037] Hajifoghaha, M. , Mirmiran, P. , & Alizadeh, S. (2016). Modification of food consumption, reduction of breast cancer: A review study. Journal of Isfahan Medical School, 34, 683–691.

[fsn32708-bib-0038] Handelman, S. K. , Romero, R. , Tarca, A. L. , Pacora, P. , Ingram, B. , Maymon, E. , Chaiworapongsa, T. , Hassan, S. S. , & Erez, O. (2019). The plasma metabolome of women in early pregnancy differs from that of non‐pregnant women. PLoS One, 14(11), e0224682. 10.1371/journal.pone.0224682 31726468 PMC6855901

[fsn32708-bib-0039] Hansen, P. (2016). Influence of dietary protein and amino acids on reproduction in dairy cows. WCDS Advances in Dairy Technology, 28, 209–216.

[fsn32708-bib-0040] Hassan, M. A. , & Killick, S. R. (2004). Negative lifestyle is associated with a significant reduction in fecundity. Fertility, 81(2), 384–392. 10.1016/j.fertnstert.2003.06.027 14967378

[fsn32708-bib-0041] Herring, C. M. , Bazer, F. W. , Johnson, G. A. , & Wu, G. (2018). Impacts of maternal dietary protein intake on fetal survival, growth, and development. Experimental Biology and Medicine, 243(6), 525–533. 10.1177/1535370218758275 29466875 PMC5882021

[fsn32708-bib-0042] Homan, G. , Davies, M. , & Norman, R. (2007). The impact of lifestyle factors on reproductive performance in the general population and those undergoing infertility treatment: A review. Human Reproduction Update, 13(3), 209–223. 10.1093/humupd/dml056 17208948

[fsn32708-bib-0043] Im Yun, J. , Gong, S. P. , Song, Y. H. , & Lee, S. T. (2013). Effects of combined antioxidant supplementation on human sperm motility and morphology during sperm manipulation in vitro. Fertility and Sterility, 100(2), 373–378. 23651626 10.1016/j.fertnstert.2013.04.015

[fsn32708-bib-0044] Imdad, A. , & Bhutta, Z. A. (2011). Effect of balanced protein energy supplementation during pregnancy on birth outcomes. BMC Public Health, 11(3), 1–9. 21501434 10.1186/1471-2458-11-S3-S17 PMC3231890

[fsn32708-bib-0045] İrez, T. , Karkada, I. R. , Dutta, S. , & Sengupta, P. (2019). Obestatin in male reproduction and infertility. Asian Pacific Journal of Reproduction, 8(5), 239–243.

[fsn32708-bib-0046] Jansson, T. (2001). Amino acid transporters in the human placenta. Pediatric Research, 49(2), 141–147. 10.1203/00006450-200102000-00003 11158505

[fsn32708-bib-0047] Jensen, T. K. , Heitmann, B. L. , Jensen, M. B. , Halldorsson, T. I. , Andersson, A.‐M. , Skakkebæk, N. E. , & Dalgård, C. (2013). High dietary intake of saturated fat is associated with reduced semen quality among 701 young Danish men from the general population. The American Journal of Clinical Nutrition, 97(2), 411–418. 10.3945/ajcn.112.042432 23269819

[fsn32708-bib-0048] Jensen, T. K. , Swan, S. , Jørgensen, N. , Toppari, J. , Redmon, B. , Punab, M. , & Sparks, A. E. (2014). Alcohol and male reproductive health: A cross‐sectional study of 8344 healthy men from Europe and the USA. Human Reproduction, 29(8), 1801–1809. 10.1093/humrep/deu118 24893607 PMC4093992

[fsn32708-bib-0049] Jensen, T. K. , Swan, S. H. , Skakkebæk, N. E. , Rasmussen, S. , & Jørgensen, N. (2010). Caffeine intake and semen quality in a population of 2,554 young Danish men. American Journal of Epidemiology, 171(8), 883–891. 10.1093/aje/kwq007 20338976

[fsn32708-bib-0050] Kalhan, S. C. (1998). Protein metabolism in pregnancy. In R. M. Cowett (Ed.), Principles of perinatal—Neonatal metabolisme (pp. 207–220). Springer.

[fsn32708-bib-0051] Karmon, A. E. , Toth, T. L. , Chiu, Y. H. , Gaskins, A. J. , Tanrikut, C. , Wright, D. L. , Hauser, R. , Chavarro, J. E. , & Earth Study Team (2017). Male caffeine and alcohol intake in relation to semen parameters and in vitro fertilization outcomes among fertility patients. Andrology, 5(2), 354–361. 28187518 10.1111/andr.12310 PMC5352521

[fsn32708-bib-0052] Kerns, K. , Zigo, M. , & Sutovsky, P. (2018). Zinc: A necessary ion for mammalian sperm fertilization competency. International Journal of Molecular Sciences, 19(12), 4097. 10.3390/ijms19124097 PMC6321397 30567310

[fsn32708-bib-0053] King, J. C. (2000). Physiology of pregnancy and nutrient metabolism. The American Journal of Clinical Nutrition, 71(5), 1218S–1225S. 10.1093/ajcn/71.5.1218s 10799394

[fsn32708-bib-0054] Kocabaş, M. , Kutluyer, F. , Ertekin, Ö. , Aksu, Ö. , & Başçınar, N. (2019). Improvement of sperm motility of *Oncorhynchus mykiss* and *Salvelinus fontinalis* by L‐tryptophan. Systems Biology in Reproductive Medicine, 65(3), 187–193. 30682894 10.1080/19396368.2019.1566414

[fsn32708-bib-0055] Kutluyer, F. , Aksu, Ö. , & Kocabaş, M. (2019). Effect of L‐tryptophan on sperm quality of tigris scraper (capoeta umbla)(pisces: Cyprinidae) after cryopreservation. Cryoletters, 40(2), 77–82. 31017607

[fsn32708-bib-0056] Lawrence, J. , Peter, R. , Baxter, G. , Robson, J. , Graham, A. , & Paterson, J. (2003). Urinary excretion of salicyluric and salicylic acids by non‐vegetarians, vegetarians, and patients taking low dose aspirin. Journal of Clinical Pathology, 56(9), 651–653. 10.1136/jcp.56.9.651 12944546 PMC1770047

[fsn32708-bib-0057] Layman, D. K. , Shiue, H. , Sather, C. , Erickson, D. J. , & Baum, J. (2003). Increased dietary protein modifies glucose and insulin homeostasis in adult women during weight loss. The Journal of Nutrition, 133(2), 405–410. 10.1093/jn/133.2.405 12566475

[fsn32708-bib-0058] Lin, G. , Wang, X. , Wu, G. , Feng, C. , Zhou, H. , Li, D. , & Wang, J. (2014). Improving amino acid nutrition to prevent intrauterine growth restriction in mammals. Amino Acids, 46(7), 1605–1623. 10.1007/s00726-014-1725-z 24658999

[fsn32708-bib-0059] Malik, V. S. , Popkin, B. M. , Bray, G. A. , Després, J.‐P. , & Hu, F. B. (2010). Sugar‐sweetened beverages, obesity, type 2 diabetes mellitus, and cardiovascular disease risk. Circulation, 121(11), 1356–1364. 10.1161/CIRCULATIONAHA.109.876185 20308626 PMC2862465

[fsn32708-bib-0060] Manning, P. , & Maggio, R. (2006). Nutritional supplements. Google Patents.

[fsn32708-bib-0061] Martin, J. C. , Zhou, S. J. , Flynn, A. C. , Malek, L. , Greco, R. , & Moran, L. (2016). The assessment of diet quality and its effects on health outcomes pre‐pregnancy and during pregnancy. Paper presented at the Seminars in reproductive medicine. 10.1055/s-0036-1571353 26886241

[fsn32708-bib-0062] Martin, P. M. , Sutherland, A. E. , & Van Winkle, L. J. (2003). Amino acid transport regulates blastocyst implantation. Biology of Reproduction, 69(4), 1101–1108. 10.1095/biolreprod.103.018010 12801981

[fsn32708-bib-0063] Martin‐Hidalgo, D. , Bragado, M. J. , Batista, A. R. , Oliveira, P. F. , & Alves, M. G. (2019). Antioxidants and male fertility: From molecular studies to clinical evidence. Antioxidants (Basel), 8(4), 89. 10.3390/antiox8040089 PMC6523199 30959797

[fsn32708-bib-0064] Mínguez‐Alarcón, L. , Chavarro, J. E. , Mendiola, J. , Roca, M. , Tanrikut, C. , Vioque, J. , Jørgensen, N. , & Torres‐Cantero, A. M. (2017). Fatty acid intake in relation to reproductive hormones and testicular volume among young healthy men. Asian Journal of Andrology, 19(2), 184. 10.4103/1008-682X.190323 27834316 PMC5312216

[fsn32708-bib-0065] Mistry, H. D. , Pipkin, F. B. , Redman, C. W. , & Poston, L. (2012). Selenium in reproductive health. American Journal of Obstetrics and Gynecology, 206(1), 21–30. 10.1016/j.ajog.2011.07.034 21963101

[fsn32708-bib-0066] Moholdt, T. , & Hawley, J. A. (2020). Maternal lifestyle interventions: Targeting preconception health. Trends in Endocrinology & Metabolism, 31, 561–569. 10.1016/j.tem.2020.03.002 32284283

[fsn32708-bib-0067] Moran, L. J. , Noakes, M. , Clifton, P. M. , Tomlinson, L. , & Norman, R. J. (2003). Dietary composition in restoring reproductive and metabolic physiology in overweight women with polycystic ovary syndrome. The Journal of Clinical Endocrinology & Metabolism, 88(2), 812–819. 10.1210/jc.2002-020815 12574218

[fsn32708-bib-0068] Muralimanoharan, S. , Maloyan, A. , & Myatt, L. (2016). Mitochondrial function and glucose metabolism in the placenta with gestational diabetes mellitus: Role of miR‐143. Clinical Science, 130(11), 931–941. 26993250 10.1042/CS20160076 PMC4918818

[fsn32708-bib-0069] National Institutes of Health (Ed.) (2008). Vitamin and mineral supplement fact sheets. Office of Dietary Supplements, National Institutes of Health.

[fsn32708-bib-0070] Ng, S. H. , Simpson, S. J. , & Simmons, L. W. (2019). Sex differences in nutrient intake can reduce the potential for sexual conflict over fitness maximization by female and male crickets. Journal of Evolutionary Biology, 32(10), 1106–1116. 10.1111/jeb.13513 31385640

[fsn32708-bib-0071] Oluwole, O. F. , Salami, S. A. , Ogunwole, E. , & Raji, Y. (2016). Implication of caffeine consumption and recovery on the reproductive functions of adult male Wistar rats. Journal of Basic and Clinical Physiology and Pharmacology, 27(5), 483–491. 10.1515/jbcpp-2015-0134 27159917

[fsn32708-bib-0072] Oriá, R. B. , de Almeida, J. Z. , Moreira, C. N. , Guerrant, R. L. , & Figueiredo, J. R. (2020). Apolipoprotein E effects on mammalian ovarian steroidogenesis and human fertility. Trends in Endocrinology & Metabolism, 31(11), 872–883. 10.1016/j.tem.2020.06.003 32684408

[fsn32708-bib-0073] Owen, D. H. , & Katz, D. F. (2005). A review of the physical and chemical properties of human semen and the formulation of a semen simulant. Journal of Andrology, 26(4), 459–469. 10.2164/jandrol.04104 15955884

[fsn32708-bib-0074] Pant, N. , Kumar, G. , Upadhyay, A. , Gupta, Y. , & Chaturvedi, P. (2015). Correlation between lead and cadmium concentration and semen quality. Andrologia, 47(8), 887–891. 25228328 10.1111/and.12342

[fsn32708-bib-0075] Peacock, M. (2010). Calcium metabolism in health and disease. Clinical Journal of the American Society of Nephrology, 5(Suppl 1), S23–S30. 10.2215/CJN.05910809 20089499

[fsn32708-bib-0076] Qazi, I. , Angel, C. , Yang, H. , Pan, B. , Zoidis, E. , Zeng, C.‐J. , Han, H. , & Zhou, G.‐B. (2018). Selenium, selenoproteins, and female reproduction: A review. Molecules, 23(12), 3053. 10.3390/molecules23123053 PMC6321086 30469536

[fsn32708-bib-0077] Ricci, E. , Al‐Beitawi, S. , Cipriani, S. , Alteri, A. , Chiaffarino, F. , Candiani, M. , Gerli, S. , Viganó, P. , & Parazzini, F. (2018). Dietary habits and semen parameters: A systematic narrative review. Andrology, 6(1), 104–116. 10.1111/andr.12452 29266782

[fsn32708-bib-0078] Rosen, C. J. , & Gallagher, J. C. (2011). The 2011 IOM report on vitamin D and calcium requirements for North America: Clinical implications for providers treating patients with low bone mineral density. Journal of Clinical Densitometry, 14(2), 79–84. 10.1016/j.jocd.2011.03.004 21787514

[fsn32708-bib-0079] Sadeu, J. , Hughes, C. L. , Agarwal, S. , & Foster, W. G. (2010). Alcohol, drugs, caffeine, tobacco, and environmental contaminant exposure: Reproductive health consequences and clinical implications. Critical Reviews in Toxicology, 40(7), 633–652. 10.3109/10408444.2010.493552 20662712

[fsn32708-bib-0081] Salas‐Huetos, A. , Bulló, M. , & Salas‐Salvadó, J. (2017). Dietary patterns, foods and nutrients in male fertility parameters and fecundability: A systematic review of observational studies. Human Reproduction Update, 23(4), 371–389. 10.1093/humupd/dmx006 28333357

[fsn32708-bib-0082] Salas‐Huetos, A. , James, E. R. , Aston, K. I. , Jenkins, T. G. , & Carrell, D. T. (2019). Diet and sperm quality: Nutrients, foods and dietary patterns. Reproductive Biology, 19(3), 219–224. 10.1016/j.repbio.2019.07.005 31375368

[fsn32708-bib-0083] Salas‐Huetos, A. , Rosique‐Esteban, N. , Becerra‐Tomás, N. , Vizmanos, B. , Bulló, M. , & Salas‐Salvadó, J. (2018). The effect of nutrients and dietary supplements on sperm quality parameters: A systematic review and meta‐analysis of randomized clinical trials. Advances in Nutrition, 9(6), 833–848. 10.1093/advances/nmy057 30462179 PMC6247182

[fsn32708-bib-0084] Sangeeta, S. , Arangasamy, A. , Kulkarni, S. , & Selvaraju, S. (2015). Role of amino acids as additives on sperm motility, plasma membrane integrity and lipid peroxidation levels at pre‐freeze and post‐thawed ram semen. Animal Reproduction Science, 161, 82–88. 10.1016/j.anireprosci.2015.08.008 26362050

[fsn32708-bib-0085] Santiago‐Moreno, J. , Bernal, B. , Pérez‐Cerezales, S. , Castaño, C. , Toledano‐Díaz, A. , Esteso, M. C. , Gutiérrez‐Adán, A. , López‐Sebastián, A. , Gil, M. G. , Woelders, H. , & Blesbois, E. (2019). Seminal plasma amino acid profile in different breeds of chicken: Role of seminal plasma on sperm cryoresistance. PLoS One, 14(1), e0209910. 10.1371/journal.pone.0209910 30608977 PMC6319765

[fsn32708-bib-0086] Santoro, N. , Polotsky, A. J. , Rieder, J. , & Kondapalli, L. A. (2019). Nutrition and reproduction. In J. F. Strauss (Ed.), Yen & Jaffe's reproductive endocrinology (pp. 447‐458.e6). Elsevier.

[fsn32708-bib-0087] Sharma, R. , Biedenharn, K. R. , Fedor, J. M. , & Agarwal, A. (2013). Lifestyle factors and reproductive health: Taking control of your fertility. Reproductive Biology and Endocrinology, 11(1), 66. 10.1186/1477-7827-11-66 23870423 PMC3717046

[fsn32708-bib-0088] Showell, M. G. , Brown, J. , Yazdani, A. , Stankiewicz, M. T. , & Hart, R. J. (2011). Antioxidants for male subfertility. Cochrane Database of Systematic Reviews, (1), Cd007411. 10.1002/14651858.CD007411.pub2 21249690

[fsn32708-bib-0089] Silva, T. , Jesus, M. , Cagigal, C. , & Silva, C. (2019). Food with influence in the sexual and reproductive health. Current Pharmaceutical Biotechnology, 20(2), 114–122. 10.2174/1389201019666180925140400 30255750

[fsn32708-bib-0090] Simopoulos, A. P. (1999). Genetic variation and nutrition. World Review of Nutrition and Dietetics, 84, 118. 10489819 10.1159/000059678

[fsn32708-bib-0091] Skoracka, K. , Eder, P. , Łykowska‐Szuber, L. , Dobrowolska, A. , & Krela‐Kaźmierczak, I. (2020). Diet and nutritional factors in male (In) fertility—Underestimated factors. Journal of Clinical Medicine, 9(5), 1400. 10.3390/jcm9051400 PMC7291266 32397485

[fsn32708-bib-0092] Solanky, K. S. , Bailey, N. J. C. , Beckwith‐Hall, B. M. , Davis, A. , Bingham, S. , Holmes, E. , Nicholson, J. K. , & Cassidy, A. (2003). Application of biofluid 1H nuclear magnetic resonance‐based metabonomic techniques for the analysis of the biochemical effects of dietary isoflavones on human plasma profile. Analytical Biochemistry, 323(2), 197–204. 10.1016/j.ab.2003.08.028 14656525

[fsn32708-bib-0093] Sørensen, L. B. , Søe, M. , Halkier, K. H. , Stigsby, B. , & Astrup, A. (2012). Effects of increased dietary protein‐to‐carbohydrate ratios in women with polycystic ovary syndrome. The American Journal of Clinical Nutrition, 95(1), 39–48. 10.3945/ajcn.111.020693 22158730

[fsn32708-bib-0094] Stamets, K. , Taylor, D. S. , Kunselman, A. , Demers, L. M. , Pelkman, C. L. , & Legro, R. S. (2004). A randomized trial of the effects of two types of short‐term hypocaloric diets on weight loss in women with polycystic ovary syndrome. Fertility and Sterility, 81(3), 630–637. 10.1016/j.fertnstert.2003.08.023 15037413

[fsn32708-bib-0095] Stephens, T. V. , Payne, M. , Ball, R. O. , Pencharz, P. B. , & Elango, R. (2015). Protein requirements of healthy pregnant women during early and late gestation are higher than current recommendations. The Journal of Nutrition, 145(1), 73–78. 10.3945/jn.114.198622 25527661

[fsn32708-bib-0096] Tan, C. H. , Denny, C. H. , Cheal, N. E. , Sniezek, J. E. , & Kanny, D. (2015). Alcohol use and binge drinking among women of childbearing age—United States, 2011–2013. MMWR. Morbidity and Mortality Weekly Report, 64(37), 1042–1046. 10.15585/mmwr.mm6437a3 26401713

[fsn32708-bib-0097] Teague, C. , Holmes, E. , Maibaum, E. , Nicholson, J. , Tang, H. , Chan, Q. , Elliott, P. , & Wilson, I. (2004). Ethyl glucoside in human urine following dietary exposure: Detection by 1H NMR spectroscopy as a result of metabonomic screening of humans. The Analyst, 129(3), 259–264. 10.1039/b314316n 14978530 PMC6556765

[fsn32708-bib-0098] Thierman, J. S. & Hallaj, I. M. Ingestible Supplement for Women. Google patent US20120040018A1.

[fsn32708-bib-0099] Tosti, V. , Bertozzi, B. , & Fontana, L. (2017). Health benefits of the mediterranean diet: Metabolic and molecular mechanisms. The Journals of Gerontology: Series A, 73(3), 318–326. 10.1093/gerona/glx227 PMC7190876 29244059

[fsn32708-bib-0100] Tremellen, K. , & Pearce, K. (2015). Nutrition, fertility, and human reproductive function. CRC Press.

[fsn32708-bib-0101] Van Tienhoven, A. (1968). Effects of nutrition on reproduction. Reprod Physiol Overteb, 355–387.

[fsn32708-bib-0102] Veaute, C. , Andreoli, M. F. , Racca, A. , Bailat, A. , Scalerandi, M. V. , Bernal, C. , & Malan Borel, I. (2007). Effects of isomeric fatty acids on reproductive parameters in mice. American Journal of Reproductive Immunology, 58(6), 487–496. 10.1111/j.1600-0897.2007.00530.x 17997747

[fsn32708-bib-0103] Villamor, E. , & Jansen, E. C. (2016). Nutritional determinants of the timing of puberty. Annual Review of Public Health, 37(1), 33–46. 10.1146/annurev-publhealth-031914-122606 26789387

[fsn32708-bib-0104] Visentin, C. E. , Masih, S. P. , Plumptre, L. , Schroder, T. H. , Sohn, K.‐J. , Ly, A. , Lausman, A. Y. , Berger, H. , Croxford, R. , Lamers, Y. , Kim, Y.‐I. , & O'Connor, D. L. (2016). Low serum vitamin B‐12 concentrations are prevalent in a cohort of pregnant Canadian women. The Journal of Nutrition, 146(5), 1035–1042. 10.3945/jn.115.226845 27075906

[fsn32708-bib-0105] Voelker, R. (2013). Even low, regular alcohol use increases the risk of dying of cancer. JAMA, 309(10), 970. 10.1001/jama.2013.2104 23483149

[fsn32708-bib-0106] Wang, J. , Wu, Z. , Li, D. , Li, N. , Dindot, S. V. , Satterfield, M. C. , Bazer, F. W. , & Wu, G. (2012). Nutrition, epigenetics, and metabolic syndrome. Antioxidants & Redox Signaling, 17(2), 282–301. 10.1089/ars.2011.4381 22044276 PMC3353821

[fsn32708-bib-0107] Watson, R. R. (2015). Handbook of fertility: Nutrition, diet, lifestyle and reproductive health. Academic Press.

[fsn32708-bib-0108] Whitfield, P. D. , German, A. J. , & Noble, P.‐J.‐ M. (2004). Metabolomics: An emerging post‐genomic tool for nutrition. British Journal of Nutrition, 92(4), 549–555. 10.1079/BJN20041243 15522124

[fsn32708-bib-0109] Whitworth, K. W. , Baird, D. D. , Stene, L. C. , Skjaerven, R. , & Longnecker, M. P. (2011). Fecundability among women with type 1 and type 2 diabetes in the Norwegian Mother and Child Cohort Study. Diabetologia, 54(3), 516–522. 10.1007/s00125-010-2003-6 21170514 PMC3650679

[fsn32708-bib-0110] Williams, C. M. (2000). Dietary fatty acids and human health. Annales De Zootechnie, 49, 165–180. 10.1051/animres:2000116

[fsn32708-bib-0111] Wilson, R. P. (2003). Amino acids and proteins. In J. E. Halver & R. W. Hardy (Eds.), Fish nutrition (pp. 143–179). Elsevier.

[fsn32708-bib-0112] Wirleitner, B. , Vanderzwalmen, P. , Stecher, A. , Spitzer, D. , Schuff, M. , Schwerda, D. , Bach, M. , Schechinger, B. , & Herbert Zech, N. (2012). Dietary supplementation of antioxidants improves semen quality of IVF patients in terms of motility, sperm count, and nuclear vacuolization. International Journal for Vitamin and Nutrition Research, 82(6), 391–398. 10.1024/0300-9831/a000136 23823924

[fsn32708-bib-0113] Wood, A. M. , Kaptoge, S. , Butterworth, A. S. , Willeit, P. , Warnakula, S. , Bolton, T. , Paige, E. , Paul, D. S. , Sweeting, M. , Burgess, S. , Bell, S. , Astle, W. , Stevens, D. , Koulman, A. , Selmer, R. M. , Verschuren, W. M. M. , Sato, S. , Njølstad, I. , Woodward, M. , … Emerging Risk Factors Collaboration/EPIC‐CVD/UK Biobank Alcohol Study Group (2018). Risk thresholds for alcohol consumption: Combined analysis of individual‐participant data for 599 912 current drinkers in 83 prospective studies. Lancet, 391(10129), 1513–1523. 29676281 10.1016/S0140-6736(18)30134-X PMC5899998

[fsn32708-bib-0114] Wu, G. (2013). Functional amino acids in nutrition and health. Amino Acids, 45, 407–411. 10.1007/s00726-013-1500-6 23595206

[fsn32708-bib-0115] Wu, G. , Bazer, F. , Datta, S. , Johnson, G. , Li, P. , Satterfield, M. , & Spencer, T. (2008). Proline metabolism in the conceptus: Implications for fetal growth and development. Amino Acids, 35(4), 691–702. 10.1007/s00726-008-0052-7 18330497

[fsn32708-bib-0116] Wu, G. , Bazer, F. W. , Satterfield, M. C. , Li, X. , Wang, X. , Johnson, G. A. , Burghardt, R. C. , Dai, Z. , Wang, J. , & Wu, Z. (2013). Impacts of arginine nutrition on embryonic and fetal development in mammals. Amino Acids, 45(2), 241–256. 10.1007/s00726-013-1515-z 23732998

[fsn32708-bib-0117] Wu, G. , Imhoff‐Kunsch, B. , & Girard, A. W. (2012). Biological mechanisms for nutritional regulation of maternal health and fetal development. Paediatric and Perinatal Epidemiology, 26, 4–26. 10.1111/j.1365-3016.2012.01291.x 22742599

[fsn32708-bib-0118] Zhu, Y. , Tian, F. , Li, H. , Zhou, Y. , Lu, J. , & Ge, Q. (2015). Profiling maternal plasma microRNA expression in early pregnancy to predict gestational diabetes mellitus. International Journal of Gynecology & Obstetrics, 130(1), 49–53. 10.1016/j.ijgo.2015.01.010 25887942

